# Prognostic value of TGF-β in lung cancer: systematic review and meta-analysis

**DOI:** 10.1186/s12885-019-5917-5

**Published:** 2019-07-15

**Authors:** Jue Li, Cheng Shen, Xin Wang, Yutian Lai, Kun Zhou, Pengfei Li, Lunxu Liu, Guowei Che

**Affiliations:** Department of Thoracic Surgery, West-China Hospital, Sichuan University, Chengdu, 610041 China

**Keywords:** TGF-β, Lung cancer, Prognosis, Meta-analysis

## Abstract

**Background:**

Lung cancer is the most important cause of cancer-related deaths worldwide and the overall survival of patients with non-small cell lung cancer has not improved. Transforming growth factor beta or TGF-β is a polypeptide member of the transforming growth factor beta superfamily of cytokines, while far fewer clinical studies addressing the association between TGF-β expression and the disease prognosis have been reported up to now. Therefore, our meta-analysis aims to determine the prognostic significance of TGF-β expression in lung cancer patients.

**Methods:**

PubMed, EMBASE, the Web of Science and China National Knowledge Infrastructure (CNKI) databases were searched for full-text literature citations. We applied the hazard ratio (HR) with 95% confidence interval (CI) as the appropriate summarized statistics. Q-test and I^2^ statistic were used to estimate the level of heterogeneity. The publication bias was detected by Begg’s test and Egger’s test.

**Results:**

Eight eligible studies involving 579 patients were selected for this meta-analysis. The combined HR for the eight eligible studies was 2.17 (95% CI: 1.71–2.77, *P* < 0.00001) and heterogeneity of overall prognosis was relatively low (I^2^ = 14.2%, *P* = 0.319). We further undertook the subgroup analysis including assessment of the association between TGF-β expression and pathology of the lung cancer, treatment and quantity of sample in studies. All the results revealed that a significantly high TGF-β expression in patients was an indicator of poor survival. Neither Begg’s test nor Egger’s test found publication bias in any analysis.

**Conclusions:**

The present evidence indicates that TGF-β expression can significantly predict the worse prognosis in patients with lung cancer. The findings of our meta-analysis may be confirmed in the future by the use of more updated review pooling and additional relevant investigations.

## Background

Lung cancer is the most common malignant tumor in the world [1]. It is still difficult to improve the overall survival of patients with non-small cell lung cancer (NSCLC) and the most patients their treatment is limited to chemotherapy or radiotherapy. Further, a simple and effective tool for predicting therapeutic efficacy and prognosticating the disease of the patients with lung cancer is lacking [2, 3]. With the development of targeted therapy research and the popularity of clinical application, many concerned researchers are exploring new biomarkers that can be used as prognostic factors for lung cancer targeted therapy.

Transforming growth factor beta (TGF-β) is a member of a multifunctional cytokine family that regulates cell proliferation [4, 5]. It appears to play a dual role in cancer. TGF-β signaling that acts as a tumor suppressor inhibits cell proliferation in normal epithelial cells and hematopoietic cells [5–8]. Studies have shown that many tumors evade immune system recognition by increasing the expression of TGF-β in the environment, thereby increasing the risk of tumor recurrence and metastasis [9]. However, fewer studies have examined the association between TGF-β expression and prognosis of patients with lung cancer. At the same time, research has shown that high TGF-βexpression predicts poor prognosis in patients with liver cancer [10]. Therefore, we performed this meta-analysis to assess the prognostic value of TGF-β in patients with NSCLC.

## Method

### Searching strategies

All of the research was published from June 1993 to July 2018 in the Pubmed, Web of Science, EMBASE and CNKI databases. The key words used are as follows:(I) “TGF-β or TGF-beta”; (II) “Pulmonary Neoplasms or Lung Cancer”; (III) “Prognoses or Prognostic Factors”. Additionally, to avoid duplication of data from different publications from the same author or research team, we further studied these articles to ensure that there was no duplication of research.

### Inclusion and exclusion criteria

Inclusion criteria: (I) Lung cancer is the main research object; (II) Demographic data or survival curves can be obtained in original studies. (III) All the study published with full texts.

Exclusion criteria: (I) The type of article that does not include a review, case report, letter and meeting report.(II) Non-human subject studies. (III) The HR value could not be obtained from the provided data.

### Quality assessment

The guideline of Newcastle-Ottawa Scale (NOS) was used for evaluating this research including three perspectives of selection, comparability and exposure. The assessment tool including the star system, a maximum of 9 stars, was used in this research. Specific evaluation system is that 8–9 stars are high quality; 6–7 stars are reasonable quality, and 6 stars or less are bad. (Table 1).

**Table 1:** Characteristics of the included studies

Study	Year	Country	Ethnicity	Design	NOS score	Sample	PE	NE	Gender (M/F)	Age (years)	Method	Stage	ADC	SCC	Others	Treatment	Follw-up(months)
Hasegawa et al.	2001	Japan	Asian	ROS	7	53	–	–	27/26	64	ELISA	I-IV	33	19	1	surgery	41(6–74)
Huang et al.	2014	China	Asian	ROS	8	194	99	95	138/56	–	FFPE	I-IV	107	76	11	surgery	60(5–72)
Kumar et al.	2011	India	Asian	ROS	8	42	–	–	–	55.5	ELISA	I-IV	–	–	–	CT	27.6(14–72)
Kumar et al.	2010	India	Asian	ROS	7	100	–	–	92/8	56	ELISA	III-IV	23	77	0	surgery+CT + RT	27(24–30)
Takanami et al.	1994	Japan	Asian	ROS	7	88	39	49	50/38	61	ELISA	I-IV	88	0	0	surgery	–
Takanami et al.	1997	Japan	Asian	ROS	7	120	66	54	–	–	ELISA	I-IV	120	0	0	surgery	84(60–144)
Xie et al.	2015	China	Asian	ROS	8	85	48	37	61/24	60	ELISA	I-IV	47	28	10	surgery+CT + RT	36(4–74)
Zhao et al.	2010	China	Asian	ROS	8	65	–	–	–	59	ELISA	III	19	42	4	CT + RT	–

*ROS* Review observational study, F*FPE* Formalin-Fixed and Parrffin-Embedded, *ELISA* Enzyme-linked immuno sorbent assay, *SCC* squamous cell carcinoma, *ADC* Adenocarcinoma, *RT* Radiation therapy, *CT* Chemotherapy, *PE* Postive expression, *NE* Negative expression

### Data collection

Two reviewers (Shen and Li) collected data from each study. Any unclear or inconsistent issues are dealt with through discussion. Excel is used to collect the following information (Table 1): author, publication year, country, study design, study period, detection method, follow-up time, included samples, age, gender, tumor staging, tumor histology, number of patients, TGF- beta expression, treatment information and survival analysis.

### Statistical analysis

The Stata 14.2 software was used to analyze the statistical data and the Engauge Digitizer 10.0 software was used to extract the survival data from the Kaplan-Meier survival curves. This study used Q test and i2 statistics to estimate the level of heterogeneity. According to the heterogeneity of I^2^ < 50% or I^2^ ≥ 50%, a fixed effect model test or a random effect model test is selected. HR > 1 implies the poor prognosis for high TGF-β level, and P < 0.05 was statistically significant. The Begg’s funnel plot analysis used to evaluate the publication bias, and the source of heterogeneity and stability of the results can be accessed by sensitivity analysis and subgroup analysis.

## Results

### The selection of included studies

**Fig. 1:**
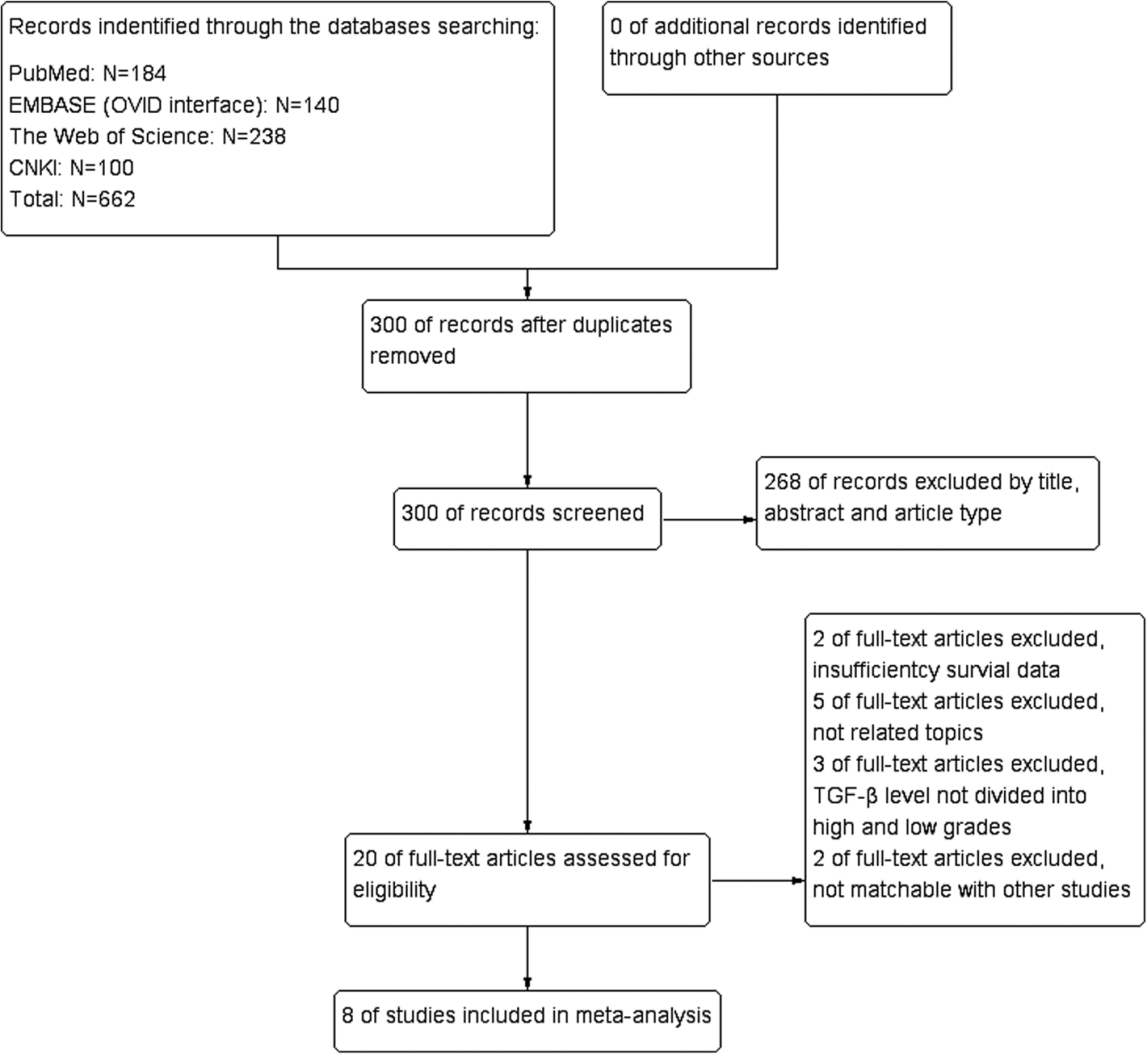
Flow chart of study selection in this meta-analysis

We searched four electronic databases including PubMed, EMBASE, Web of Science and CNKI and total number of citation is 588. There were 28 articles included by screening of titles and abstracts and excluding duplicate literature entries. Later, there were 268 papers excluded due to unqualified document types, the full text of the remaining documents was read fully. In our meta-analysis, 20 potential qualified papers were identified. Ultimately, only eight articles were involved in the present study [4, 8, 9, 11–16] (Fig. 1).

### The characteristics of included studies

The basic characteristics of eight qualified literature sources are recorded in Table 1. Takanami et al. [8, 9] focused on TGF-β expression in pulmonary adenocarcinomas. Hasegawa et al. [15] enrolled a total of 53 NSCLC tissue samples (19 squamous cell carcinomas, 33 adenocarcinomas, and 1 adenosquamous cell carcinoma) in their study. Kumar et al. [17] estimated diagnosed and untreated Indian lung cancer patients with advanced-stage NSCLC. All of the patients were confirmed by the histological examinations during fibreoptic bronchoscopy or with a computed tomography (CT) - guided procedure. The aim of the study was analyzing the efficacy of plasma TGF-β levels as a prognostic factor for survival in patients with chemotherapy. Zhao et al. [16] reported TGF-β expression in patients with stage IIIA or IIIB NSCLC treated with radiotherapy. The study of Huang et al. [10] explored the expression intensity and prognostic significance of TGF-β in NSCLC patients with immunohistochemistry outcomes. Xie et al. [4] reported the prognostic values of TGF-β in lung cancer patients in China.

### Meta-analysis results

**Fig. 2:**
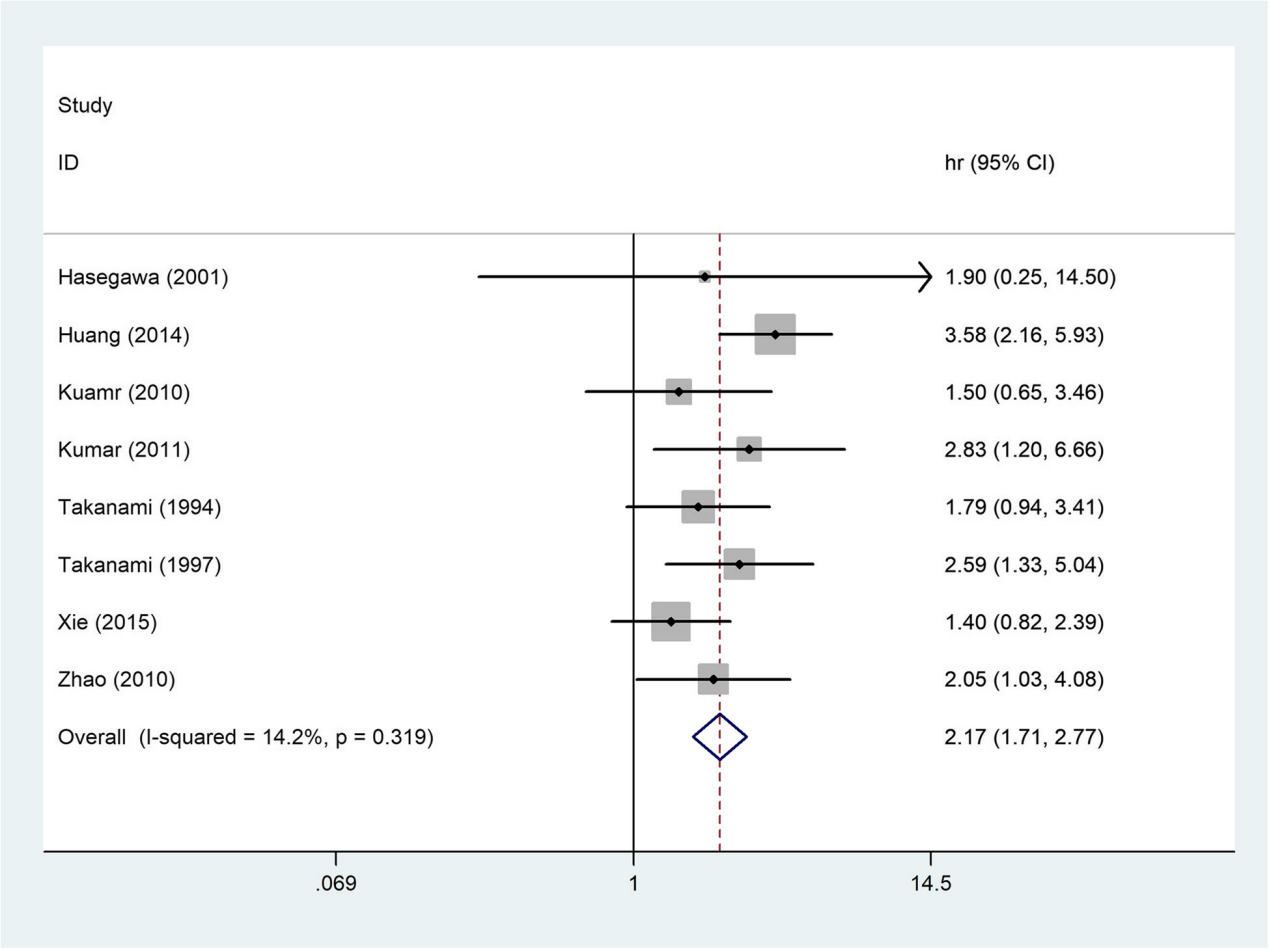
Forest plots of the eight evaluable studies assessing the prognostic value of TGF-β in lung cancer. *CI* Confidence intervals, *HR* Hazard ratio

The combined HR for the eight appropriate studies was 2.17 (95% CI: 1.71–2.77, *P* < 0.00001), suggesting that TGF-β overexpression was an indicator of poor survival for NSCLC patients (Fig. 2). Heterogeneity of overall prognosis was relatively low (I2 = 14.2%, *P* = 0.319). We further commenced the subgroup investigation.

### Valuation of the link between TGF-β expression and pathology of the lung cancer

**Fig. 3:**
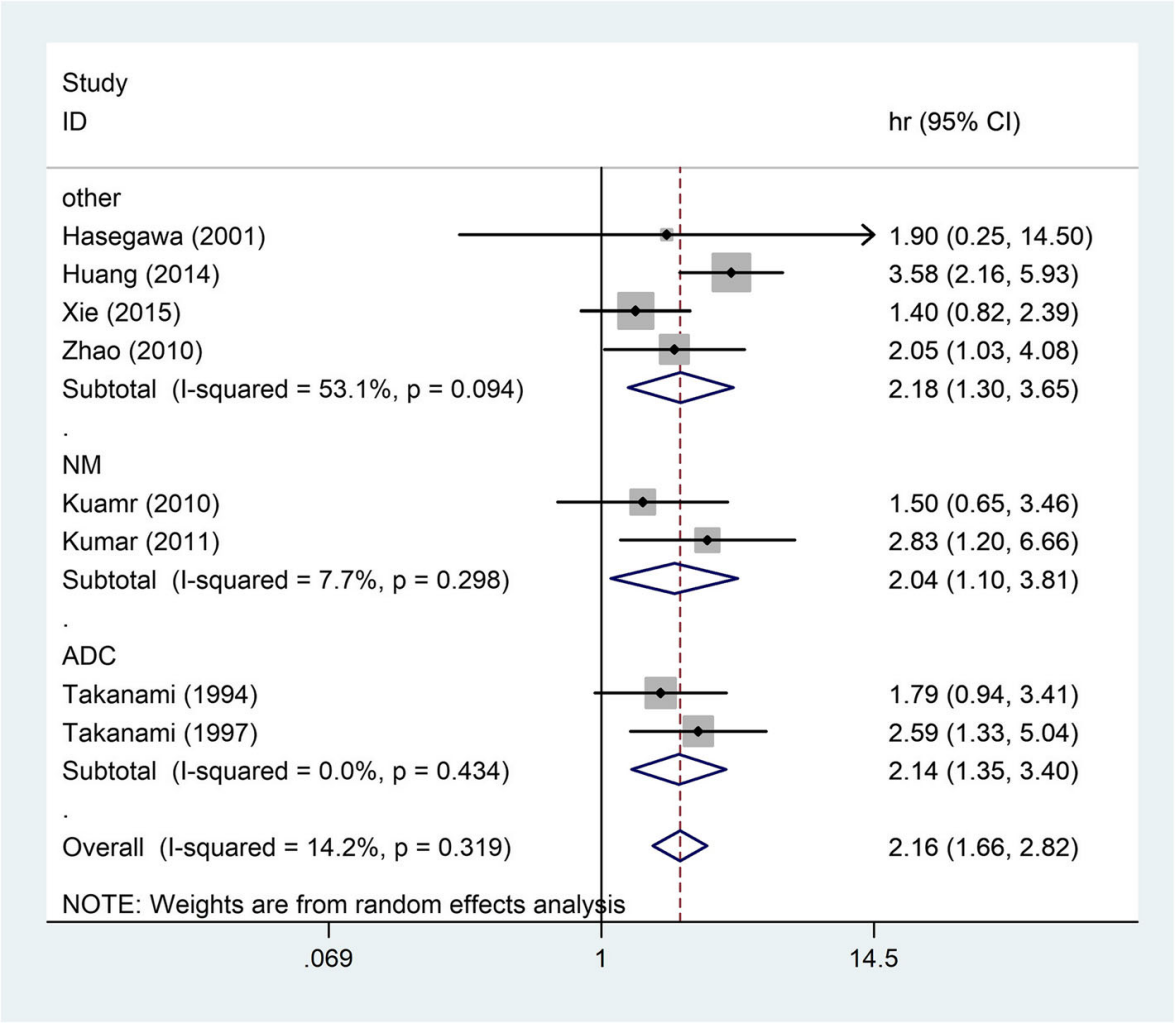
Pooled HRs for assessing of the association between TGF-β expression and pathology of the lung cancer. HR, hazard ratio, *CI* Confidence interval, *ADC* Adenocarcinoma, *NM* Not mentioned, *ES* Effect size

There were two studies reporting lung adenocarcinoma of patients and the defined HR based on this study was 2.14 (95% CI: 1.35–3.40) (Fig. 3). The fixed-effect model was then considered for this part (I^2^ = 0.0%, *P* = 0.434). Another two studies that did not define the tumor pathology showed that TGF-β overexpression might be a strong predictor of poor survival for NSCLC patients (HR = 2.04, 95% CI: 1.12–3.72) (Fig. 3). From four of the included studies patients with other pathologies of lung cancer indicated that high TGF-β expression was a marker of poor survival (HR = 2.23, 95% CI: 1.62–3.07) (Fig. 3).

### Valuation of the link between TGF-β expression and treatment

**Fig. 4:**
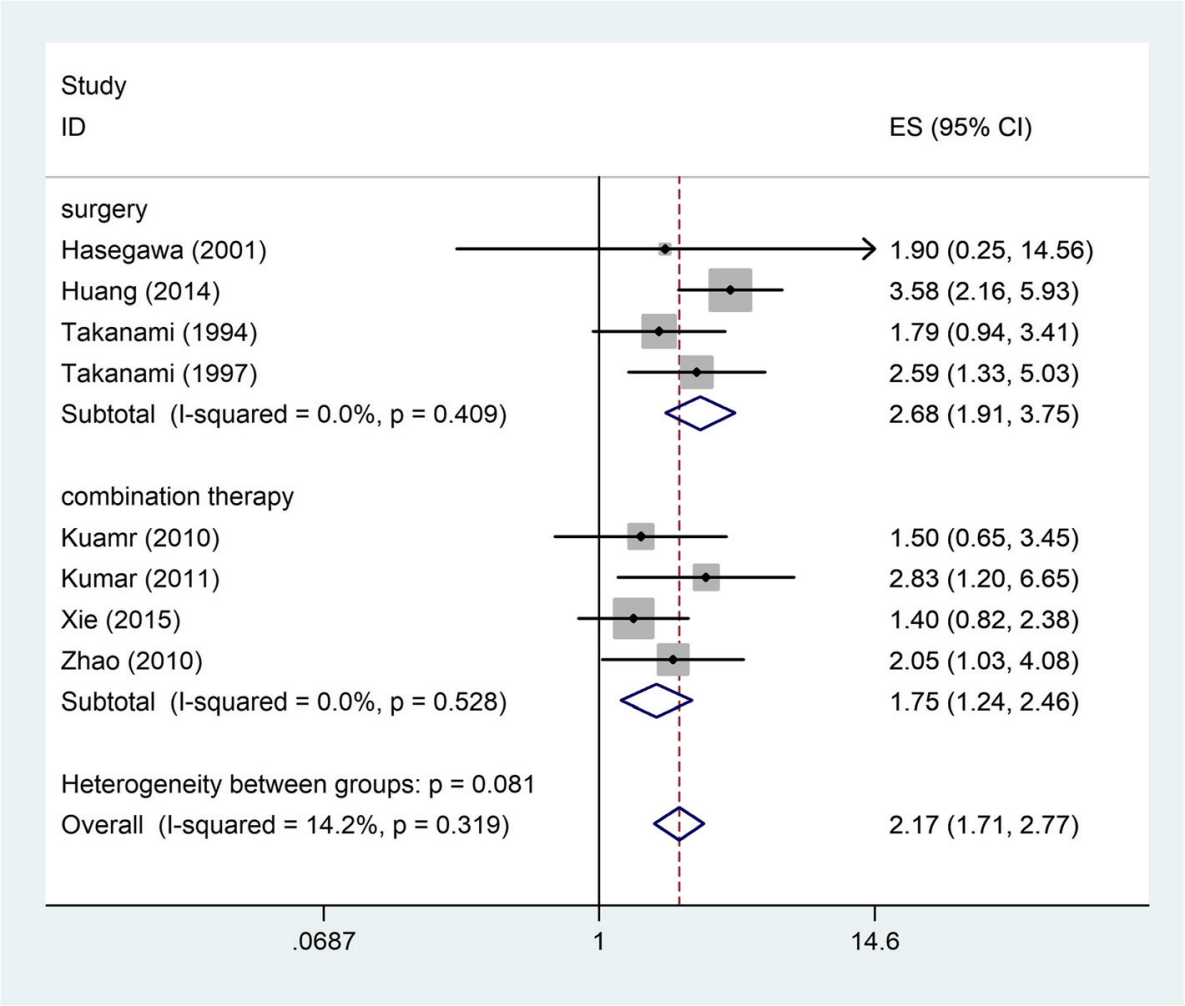
Pooled HRs for assessing of the association between TGF-β expression and treatment. *HR* Hazard ratio, *CI* Confidence interval, *ES* Effect size

We measured the association between TGF-β expression and treatment. The samples were treated only with surgery in four studies, the summarized HR of analysis was 2.68 (95% CI: 1.91–3.75) (Fig. 4). When we compared the relationship between TGF-β expression and combination therapy, it was statistically meaningful statistics (HR = 1.75; 95% CI: 1.24–2.46). These two findings showed that excessive TGF-β expression was a sign of poor survival.

### Valuation of the link between TGF-β expression and quantity of sample in studies

**Fig. 5:**
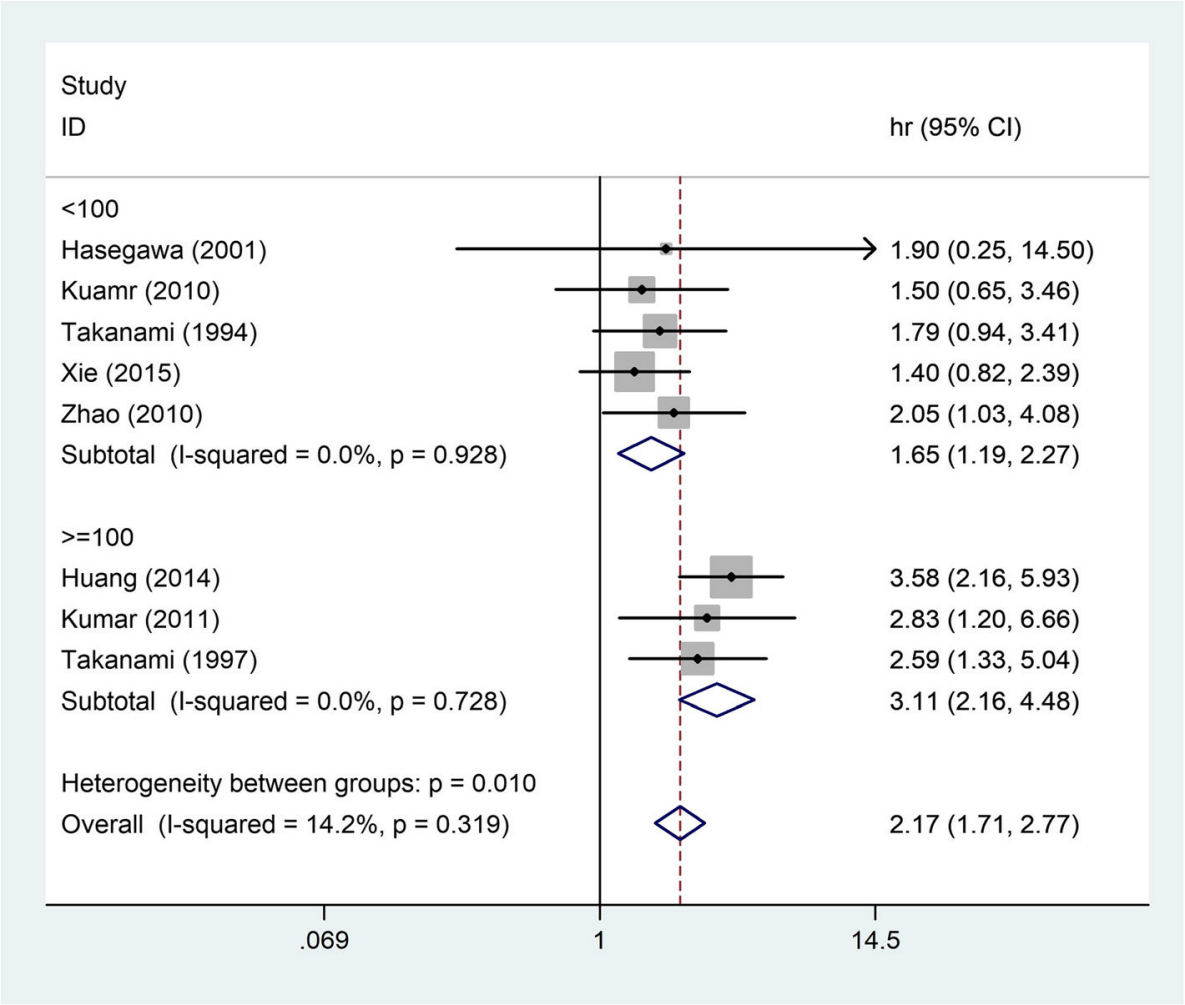
Pooled HRs for assessing of the association between TGF-β expression and quantity of sample in studies. *HR* Hazard ratio, *CI* Confidence interval, *ES* Effect size

On the one hand, with the sample greater or equal to 100, the HR was 3.11 (95% CI: 0.77–1.50) (Fig. 5). On the other hand, with the sample less than 100, the HR based on analysis also revealed the meaningfully high TGF-β expression in patients was an indicator of poor survival (HR: 1.65; 95% CI: 1.19–2.27) (Fig. 5).

### Publication bias

**Fig. 6:**
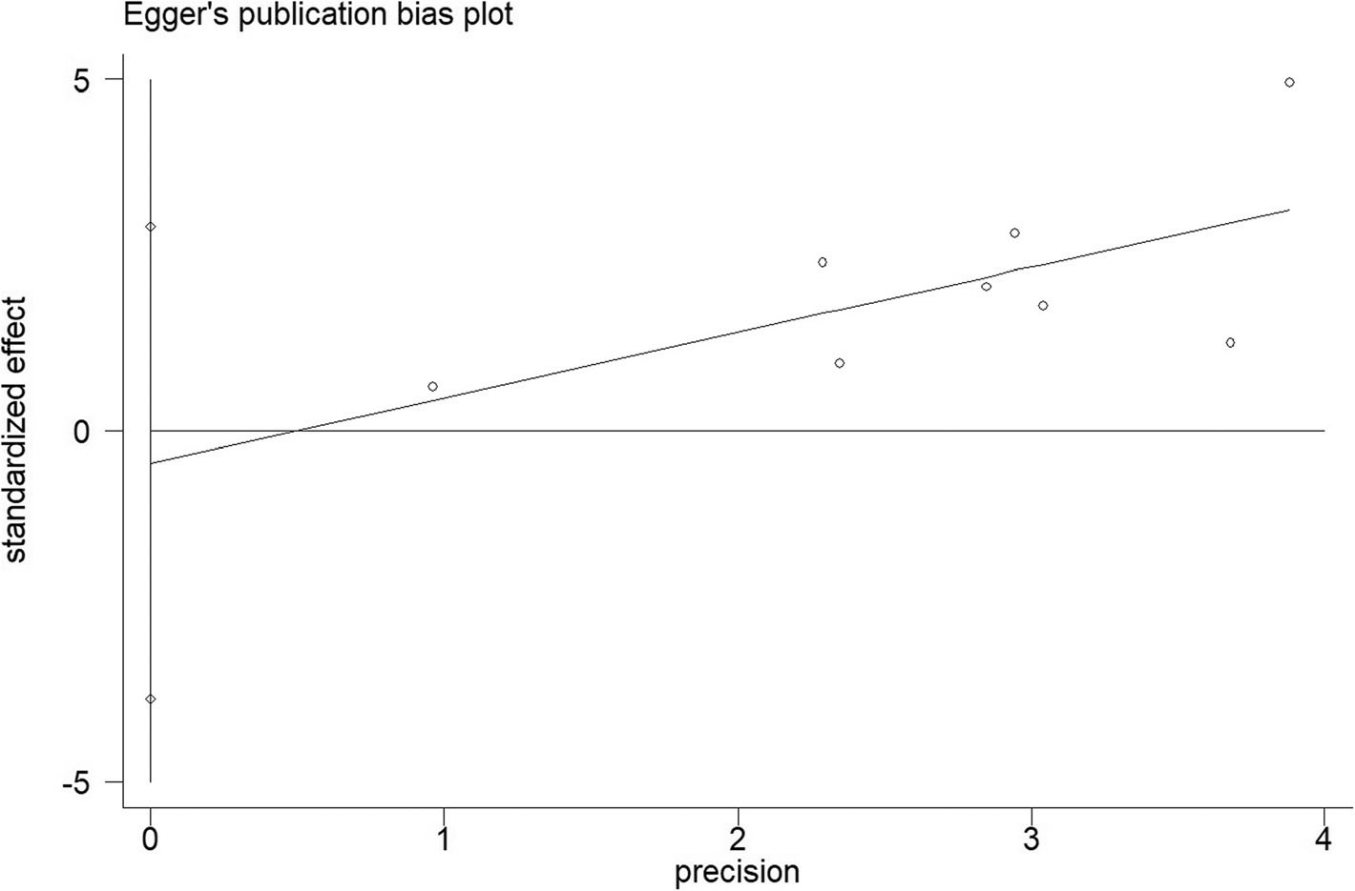
Egger’s publication bias plot

**Fig. 7:**
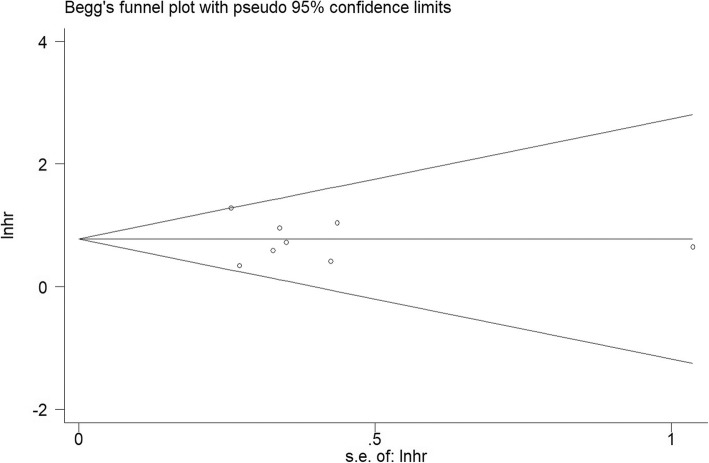
Funnel plots of publication bias on the correlation between TGF-β expression and overall survival

Both the Begg’s funnel plot and Egger’s test were completed to assess the publication bias in the literature (Figs. 6 and 7). All eight qualified studies in the literature are available generated a Begg’s test score of *P* = 0.902 and Egger’s test score of *P* = 0.747. According to the Begg’s tests result, there was no meaning publication bias detected in overall survival. Nonparametric pruning and filling methods were used to assess the impact of this bias and summary HR on overall survival and there was no meaningful publication bias in the study and the combined HR remained significant. In short, the outcomes in our research are statistically dependable.

## Discussion

To our best knowledge, this meta-analysis is the first meta-analysis to evaluate the prognostic value of TGF-β expression in patients with NSCLC, although only eight qualified literatures are available at present.

In several studies, TGF-β protein expression in patients with NSCLC has been described to correlate with survival [1, 13, 14, 18]. TGF-β appears to be involved in the tumorigenesis of the patients in these studies. The higher the expression of TGF-β protein, the more advanced tumor stage for the patients [12, 18, 19]. In addition, patients appear to be more likely to be diagnosed with lymph node metastasis if the TGF-β protein expression was higher than normal levels [5, 20].

Some studies indicate that the matrix formed by TGF-β may provide a good environment for the tumor growth, and this is a key role in the occurrence and development of cancers [5]. Fibroblasts and mononuclear cells surrounding tumor cells will contribute and produce TGF-β at the same time. It is well known that TGF-β is one of the growth factors that regulate the composition of the extracellular matrix of the alveolar epithelium and induce epithelial to mesenchymal transition (EMT). The feature including a top surface and a basal plane is described as the “polarity” of epithelial cells. The arrangement between epithelial cells prevents them from detaching from the tissue. Epithelial cells progressively lose cell polarity and adhesions, gain invasive and migratory ability, and produce extracellular matrix components with the influence of some factors during EMT [21–23]. During EMT, cancer cells lose cell-cell adhesion junctions and change into fibroblast-like morphology, resulting in superior mobility and invasiveness [24, 25]. TGF-β expression may promote the growth and differentiation of tumor cells through autocrine or paracrine activity, ultimately resulting in increased cell matrix interaction, inhibition of immune surveillance, or increased angiogenic activity [26]. TGF-β can inhibit the growth of normal epithelial cells, but tumor cells have strong resistance to TGF-β inhibition. Detection of TGF-β gene, mRNA and protein in lung cancer cell lines indicated that tumor cells have a strong proliferative response to TGF-β [27, 28]. Previous studies have observed a strong correlation between the increased TGF-β levels and the disease status in lung cancer patients without examining the prognosis. Analyzing the prognostic value of TGF-β specifically in lung cancer using meta-analysis in our study, we further performed the subgroup analysis including the assessment of the association between TGF-β expression and pathology, treatment or the quantity of sample in all studies. High expression of TGF-β was associated with worse survival in patients with NSCLC can be concluded from our results.

There are several limitations in this meta-analysis. Firstly, the number of studies included and the simple scale were relatively small. Secondly, some of the HRs with 95% CI was not directly extracted from the studies and some errors may have occurred during the rebuilding of HR calculated via Kaplan-Meier survival curves. Thirdly, all of the populations included in the eight studies evaluated in our analysis were Asian and there might be an ethnic bias. Fourthly, all included studies used ELISA to detect the TGF-β; there may be differences in reagents, operation steps and results, which may give rise to bias. The results of subgroup analysis revealed that sample size, specimen type, source of HR and survival analysis mode might contribute to the heterogeneity.

## Conclusions

In conclusion, our meta-analysis showed that high expression of TGF-beta could significantly predict poor prognosis in NSCLC patients. The small numbers of studies examined may influence the validity of this conclusion. Therefore, the results of our meta-analysis should be better confirmed with additional relevant research in the future using updated analyses.

## Data Availability

All data for this study are publicly available and are ready for the public to download at no cost from the official websites of the PubMed, EMBASE, the Web of Science and China National Knowledge Infrastructure. There is no need to have the formal permission to use data for this study. The sources and data robustness have been described in the section of “Methods”.

## Abbreviations

ADC: Adenocarcinoma
CI: Confidence interval
CNKI: China National Knowledge Infrastructure
CT: Computed tomography
ELISA: Enzyme-linked immuno sorbent assay
EMT: Epithelial to mesenchymal transition
FFPE: Formalin-Fixed and Parrffin-Embedded
HR: Hazard ratio
NE: Negative expression
NOS: Newcastle-Ottawa Scale
NSCLC: Non-small cell lung cancer
PE: Postive expression
ROS: Review observational study
RT: Radiation therapy
SCC: Squamous cell carcinoma
TGF-β: Transforming growth factor beta
TNM: Tumor, Lymph Node, Metastasis
